# Porencephaly and psychosis: a case report and review of the literature

**DOI:** 10.1186/1471-244X-10-19

**Published:** 2010-03-02

**Authors:** Athanassios Douzenis, Emmanouil N Rizos, Athanasia Papadopoulou, Matilda Papathanasiou, Lefteris Lykouras

**Affiliations:** 1 2nd Department of Psychiatry, University of Athens Medical School, "Attikon" General Hospital, 1 Rimini st Athens 12462, Greece; 2 2nd Department of Radiology, University of Athens Medical School, "Attikon" General Hospital, 1 Rimini st Athens 12462, Greece

## Abstract

**Background:**

Malformations of the cerebral cortex are often associated with developmental delay and psychoses. Porencephaly is a rare congenital disorder of central nervous system involving a cyst or a cavity filled with cerebrospinal fluid, in brain's parenchyma.

**Case presentation:**

We present a 25 years old woman with her first psychotic episode. She also suffers from porencephaly in the frontotemporal lobes region. It is emphasized that the two consistently abnormal brain regions in schizophrenia research had significant damage in this patient since birth. There is a total of only five cases of schizencephaly or porencephaly associated with psychosis in the scientific literature. Their clinical characteristics as well as the imaging results are described.

**Conclusion:**

It is unclear if porencephaly and psychosis concur by chance or are causally related. The area where the porencephalic cysts appear seems to be of relevance. This case highlights the need for further research.

## Background

Malformations of the cerebral cortex are often associated with developmental delay and psychoses. Porencephaly is an extremely rare congenital disorder of the central nervous system involving a cyst or a cavity filled with cerebrospinal fluid, in the brain's parenchyma. It is caused by either local damage from ischemia in the brain hemisphere, or most commonly, hemorrhage after birth. It can also occurs as a consequence of abnormal development before birth (these are less common cases) [1,2].

Congenital brain lesions include two types of porencephaly: genetic porencephaly: Genetic porencephaly resulting from maldevelopment during early neuronal migration and encephalophaloplastic porencephaly, which is late prenatal or perinatal vascular lesion due to arterial ischemic stroke or venous thrombosis. Porencephalic cysts can be located in any lobe or lobes of the two brain hemispheres [3].

Prefrontal cortex and limbic system hypotheses are the predominant neuroanatomical hypotheses of psychosis. Decreased prefrontal gray or white matter volumes, or disturbed prefrontal metabolism and blood flow. As well as decreased hippocampal and entothinal cortex volume, in psychotic patients, have been demonstrated. All these are strong indications that prefrontal cortical and medial temporal regions are associated with psychosis [4-6].

## Case Presentation

A 25-year-old woman presented at the emergency department of the 2^nd ^Psychiatry Department in Attikon University General Hospital with psychotic symptoms. She was accompanied by her mother and brother. There was no previous psychiatric history. During the last three days before admission, she was frightened, couldn't sleep properly and said that people talked to her through the television set. On the day of her admission, she stayed home, closed the windows and frequently called her husband at work, asking for help, because something "very bad" would happen. Her husband tried to calm her, but that was impossible. When he arrived home she was lying in bed and didn't offer any explanations for her behavior.

On mental state examination, the patient was oriented in person, place and time. She expressed persecutory delusions that were related to violent incidences (riots) that occurred the previous days in Athens. Her thought content was characterized by delusions of persecution and reference. Her speech was interrupted and she appeared to experience thought blocking. She did not admit to auditory hallucinations. Patient's affect was subjectively anxious and fearful but objectively her affect **was **blunted and she did not display any insight into her condition. She had psychomotor retardation and her speech was slow. She was not suicidal. She told us she suffered from insomnia the last days before admission and she was co-operative, accepting admission. Upon admission PANSS scores were: Positive: +17, Negative:-22, General Psychopathology: 35 Neurological examination revealed right arm weakness (proximal 3|5, distal 2+/5 according to the MRC scale of evaluation), pyramidal type rigidity with increase of deep tendon reflexes, muscle atrophy and stereoagnosia of the same arm. The Mini-Mental State Examination was normal (30/30). A neuropsychological examination showed few alterations, with impairment of verbal memory, attention and ability to plan (WAIS: 64, Verbal: 69, Performance: 63).

She had no past psychiatric history or family psychiatric history. According to her medical history, she suffers from porencephaly located in the front-temporal lobes region since birth. The patient had no speech development problems or severe mental retardation that is often associated with porencephaly. However, she had a slight spastic paresis in her right arm. The patient never received antiepileptic treatment and no physiotherapy was ever suggested for her arm spasticity and movement difficulties. According to the relatives, neurosurgery assessment was never suggested to them. When inpatient a referral to the neurosurgeons was made but after inspecting her brain MRI and bearing in mind that there were no clinical signs of increased CSF pressure it was felt that no further assessment was needed. Generally, treatment of porencephaly aims at alleviating symptoms as there is no treatment to induce brain growth in the missing sections and that can include antiepileptic medication, physiotherapy or a shunt to remove excess cerebrospinal fluid [7].

Haematological and biochemistry blood test results, thyroid tests, toxicological tests, gonathotropins and cortizol levels were within normal range and did not reveal any other related medical condition.

The patient was not very social as a child; she had only few friends, however she managed to complete secondary education. She grew up in a small village and lived there up until she was 23. She had no intimate relationships until she met his husband and left with him for Athens. She kept their house but her husband did all external jobs and she never left home on her own. Few days before her illness, there was an argument and the husband threatened to leave.

**Figure 1:**
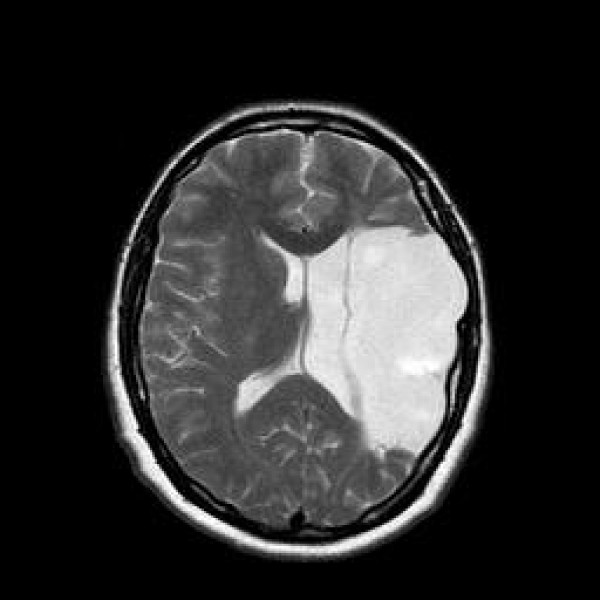
**Coronal T1 Large area of porencephaly in the left frontal and parietal lobes, in the anatomic distribution of left middle cerebral artery branches**. Wallerian degeneration of the ipsilateral cerebral peduncle was also present (not shown).

**Figure 2:**
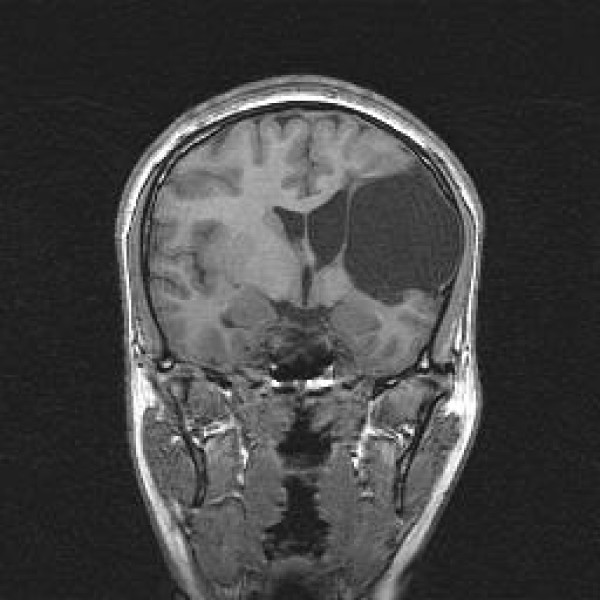
**Axial T2 Large area of porencephaly in the left frontal and parietal lobes with compensatory dilatation of the lateral ventricle**.

A brain MRI was performed (fig 1 and fig 2) in order to get a more detailed image of her brain. It confirmed the nature of the lesion and revealed the existence of a large porencephalic cyst on the left frontal and temporal lobes. The examination revealed also atrophy in the left side of brainstem due to wallerian degeneration. This radiographic finding is indicative of an old and non progressive lesion

Antipsychotic agents which are strong blockers of D2 receptors were excluded because of her possible vulnerability to extrapyramidal symptoms due to the brain damage. Bearing in mind she was underweight, she was started on olanzapine 10 mg, which has been used successfully for the treatment of another patient with porencephaly and psychosis according to the literature [8]. The psychotic symptoms improved progressively during her inpatient stay. It was clear that there was a significant improvement in relation to the delusional persecutory ideas and a complete remission of these symptoms was achieved. PANSS (Positive: +10, Negative:-18, General Psychopathology: 19). Upon discharge there were no signs of psychomotor retardation The patient was discharged after a 2-week inpatient stay and received follow-up outpatient care with psychiatric appointments). After one year of follow-up, psychiatrically she is table and maintains contact; she continues her treatment and is now receiving 5 mgr of Olanzapine. The diagnosis is schizophrenia paranoid type or psychotic disorder due to a general medical condition. (DSM-IV TR)[9].

The present case had clinical manifestation of porencephaly, i.e. mild mental retardation and spastic paresis in her left arm accompanied by motor impairment. However, this patient did not have speech development problems or seizures. The location of porencephalic cyst, in frontal and temporal lobes, can be associated with psychotic features, or one can suggest that damaged brain regions possibly triggered the patient's potential vulnerability to psychotic symptoms

We reviewed the scientific literature for other cases with porencephaly and psychosis to investigate the above hypothesis. Pae et al. in 2009 report the case of a patient who developed a psychotic episode, possibly, associated with multiple leukoencephalomalacia and Porencephaly changes in the brain cortex. In this case, a 30-year-old woman had persecutory ideas and visual hallucination. Multiple focal leukoencephalomalacia changes in the left frontal lobe, bilateral occipital lobes and left basal ganglia, and a large porencephalic change in the right temporal lobe were found in with (MRI) [8].

Schizencephaly is also a rare congenital neurodevelopment disorder of brain cortex which is characterized by abnormal slits or clefts in the cerebral hemispheres. That malformation is a form of porencephaly. It appears with paresis, mental retardation and seizures, too [10]. Alexander et al reported two cases with schizencephaly associated with psychosis. The first patient had seizures, mental retardation and psychotic features like delusions and auditory hallucinations. The second patient had monoplegia, mental retardation and delusions[11]. Relan et al presented a case with unilateral schizencephaly cleft associated with biopolar disorder. This patient was overactive and oversocial. She also had mild motor impairment, mental retardation and presented with persecutory ideation and auditory hallucinations [12].

Cysts on the left temporal lobe have been associated with psychosis. Frontal and especially temporal lobe structure and fuction are consistently implicated in radiological imaging literature (brain imaging research) in schizophrenia [13]. In several MRI studies, medial temporal lobe structures, which include the amygdale, hippocampus, parahipoccampal gyrus and superior temporal gyrus, perform a lot of abnormalities [14]. Especially, deficit in left medial temporal lobe volume is one of the most frequent finding of Morfometry studies in schizophrenia [15]. Alves da Silva et al described the case of a patient with an arachnoid cyst in the left temporal lobe. He had psychotic symptoms such as delusions and hallucinations [16]. Vakis et al reported a case of young woman with a left temporal lobe arachnoid cyst who presented with a psychosis-like syndrome [17].

Overall, there was only one case of porencephaly and psychosis and three cases of schizencephaly (which is considered to be a variation of porencephaly) and psychosis. Table 1 summarizes these findings.

**Table 1:** Porencephaly and schizencephaly cases in the literature

		Number of cases	Anatomical Damages	Diagnosis
Porencephaly case	Pae CU., Kim JH. 2009	1	Multiple focal leukoencephalomalacia changes in the left frontal lobe, bilateral occipital lobes and left basal ganglia, and a large porencephalic change in the right temporal lobe	Psychosis
Schizencephaly cases	Alexander RC et al 1997	2	unilateral schizencephaly cleft and bilateral schizencephaly cleft	Psychosis
	Relan P et al 2002	1	unilateral schizencephaly cleft	Bipolar Affective Disorder
Current case	Douzenis et al 2009	1	a large porencephalic cyst on the left frontal and temporal lobes.	Psychosis

## Conclusion

It is still unclear whether porencephaly and psychosis concur by chance in this case or that there is actually an increased risk for psychosis in patients with porencephaly.

Our review showed that there are only two cases with porencephalic cysts and psychosis as well as 3 cases of schizencephaly. The damaged brain regions in these cases are associated with psychotic symptoms. The presence of neurodevelopment anomalies may have pathoplastic effect on the manifestation of psychosis. We hypothesize that this patient's brain lesions have a crucial influence on her vulnerability to psychotic symptoms. One can assume that even old lesions on the left frontal and temporal lobes can be an additional risk factor for the development of the disorder. She had no past or family psychiatric history and this was her first psychotic episode. Another vulnerability factor is her IQ which was 64, indicating mild mental retardation. Low IQ is associated with a higher incidence of psychotic disorder, than in normal population [18]. This patient's porencephalic cyst extended from left frontal to the left temporal lobe. Lesions in these areas have been associated with psychosis. This case highlights the need that in patients with similar clinical features as described above, radiological examination should be carried out so as to discover damaged brain lesions.

## Consent

Written informed consent was obtained from the patient in this case report.

## Competing interests

The authors declare that they have no competing interests.

## Authors' contributions

AD designed the study, wrote the article and supervised the data collection. ENR contributed to the clinical and rating evalution of the patient and to the writing of the manuscript. AP rewiewed the existing literature and contributed to article's writing. MP evaluated the radiographic findings of the brain MRI. LL had the overall supervision of the study. All authors read and approved the final manuscript.

## Pre-publication history

The pre-publication history for this paper can be accessed here:

http://www.biomedcentral.com/1471-244X/10/19/prepub

## References

[B1] Ho SS Kuzniesky RI Gilliam F Faught E Bebin M Morawetz R Congenital porencephaly:MR features and relationship to hippocampal sclerosis AJNR Am J Neuroradiol 1998 19 1 135 141 9432171 PMC8337343

[B2] Stevenson RE Hall JG Porencephaly Human Malformations and related Anomalies 2006 2 Oxford University Press 645 654

[B3] Tonni G Ferrari B Defelice C Gentini G Neonatal porencephaly in very low birth weight infants: Ultrasound timing of asphyxial injury and neurodevelopmental outcome at two years of age The Journal of Maternal-Fetal and Neonatal Medicine 2005 18 6 361 365 10.1080/14767050400029574 16390800

[B4] Buchanan RW Carpenter WT Schizofrenia and Other Psychotic Disorders Kaplan & Sadock's Comprenhesive Textbook of Psychiatry 2005 2 Lippincott Williams & Wilkins 1329 1371

[B5] Saugstad LF What is psychosis and where is located? Eur Arch Psychiatry Clin Neurosci 2008 258 Suppl 111 117 10.1007/s00406-008-2014-1 18516523

[B6] Wood J Pantelis C Velakoulis D Yucel M Fornito A McGorry P Progressive Changes in the Development Toward Schizophrenia: Studies in Subjects at Increased Symptomatic Risk Schizophrenia Bulletin 2008 34 2 322 329 10.1093/schbul/sbm149 18199631 PMC2632412

[B7] Graham David I Peter Lantos L Greenfield's Neuropathology 1997 6 Bath, UK:Arnold

[B8] Pae CU Kim JH The Leukoencephalomalacia and Porencephalia Changes in the Brain and the Potential Usefulness of Olanzapine Treatment CNS Spectr 2009 13 10 e1

[B9] American Psychiatric Association Diagnostic and Statistical Manual of Mental Disorders 2000 Fourth TR.Washington, D.C.: American Psychiatric Press, Inc

[B10] Packard AM Miller VS Delgado MR Schizenchephaly: correlations of clinical and radiological features Neurology 1997 48 1427 1434 9153485 10.1212/wnl.48.5.1427

[B11] Alexander RC Patkar AA Lapointe JS Flynn SW Honer W Schizencephaly associated with psychosis J Neurol Neurosurg Psychiatry 1997 63 373 375 10.1136/jnnp.63.3.373 9328256 PMC2169691

[B12] Relan P Chatuverdi SK Shetty B Schizencephaly Associated with Bipolar Affective Disorder Neurology India 2002 50 2 194 197 12134187

[B13] Lawrie SM McIntosh AM Hall J Owens DGC Johnstone EC Brain Stucture and Function Changes during the development of Schizophrenia: The evidence from studies of subjects at increased genetic risk Schizophrenia Bulletin 2008 34 2 330 340 10.1093/schbul/sbm158 18227083 PMC2632417

[B14] Shenton ME Dickey CC Frumin M McCarley RC A review or MRI findings in schizophrenia Schizophrenia Research 2001 49 1 52 10.1016/S0920-9964(01)00163-3 11343862 PMC2812015

[B15] Honea R Crow T Passingham D Mackay CE Regional Deficits in Brain Volume in Schizophrenia; A Meta-Analysis of Voxel -Based Morphometry Studies Am J Psychiatry 2005 162 2233 2245 10.1176/appi.ajp.162.12.2233 16330585

[B16] Alves Da Silva J Alves A Talina M Carreiro S Guimaraes J Xavier M Arachnoid cyst in a patient psychosis: a case report Annals of General Psychiatry 2007 6 167 10.1186/1744-859X-6-16 PMC1933420 17598903

[B17] Vakis AF Koutenakis DI Karabetsos DA Kalostos GN Psychosis-like syndrome associated with intermittent intracranial hypertension caused by a large arachnoid cyst of the left temporal lobe Br J Neurosurg 2006 20 3 156 9 10.1080/02688690600776986 16801049

[B18] Cooper SA Smiley E Morrison J Allan L Williamson A Finlayson J Jackson A Psychosis and adults with intellectual disabilities. Prevalence, incidence and related factors Soc Psychiatry Psychiatr Epidemiol 2007 42 7 530 536 10.1007/s00127-007-0197-9 17502974

